# Comparative Analysis of Metabolic Variations, Antioxidant Profiles and Antimicrobial Activity of *Salvia hispanica* (Chia) Seed, Sprout, Leaf, Flower, Root and Herb Extracts

**DOI:** 10.3390/molecules28062728

**Published:** 2023-03-17

**Authors:** Sara Motyka, Barbara Kusznierewicz, Halina Ekiert, Izabela Korona-Głowniak, Agnieszka Szopa

**Affiliations:** 1 Chair and Department of Pharmaceutical Botany, Faculty of Pharmacy, Collegium Medicum, Jagiellonian University, 9 Medyczna St., 30-688 Kraków, Poland; sara.motyka@doctoral.uj.edu.pl (S.M.); halina.ekiert@uj.edu.pl (H.E.); 2 Doctoral School of Medical and Health Sciences, Collegium Medicum, Jagiellonian University, 16 Św. Łazarza St., 30-530 Kraków, Poland; 3 Department of Chemistry, Technology and Biotechnology of Food, Faculty of Chemistry, Gdańsk University of Technology, 11/12 Narutowicza St., 80-233 Gdańsk, Poland; 4 Department of Pharmaceutical Microbiology, Medical Univeristy of Lublin, Chodźki 1 St., 20-093 Lublin, Poland; izabela.korona-glowniak@umlub.pl

**Keywords:** *Salvia hispanica*, chia, phytochemical analysis, antioxidant activity, antibacterial activity, antifungal activity, rosmarinic acid, Q-Orbitrap-HRMS

## Abstract

The purpose of this study was to evaluate the phytochemical profiles of the seeds, sprouts, leaves, flowers, roots and herb of *Salvia hispanica* and to demonstrate their significant contribution to antioxidant and antimicrobial activities. Applied methods were: HPLC-DAD coupled with post-column derivatization with ABTS reagent, untargeted metabolomics performed by LC-Q-Orbitrap HRMS, and two-fold micro-dilution broth method, which involved suspending a solution of tested compounds dissolved in DMSO in Mueller–Hinton broth for bacteria or Mueller–Hinton broth with 2% glucose for fungi. Metabolomic profiling using LC-Q-Orbitrap HRMS used in this study yielded the identification and preliminary characterization of one hundred fifteen compounds. The dominant class of compounds was terpenoids (31 compounds), followed by flavonoids (21 compounds), phenolic acids and derivatives (19 compounds), organic acids (16 compounds) and others (fatty acids, sugars and unidentified compounds). The organic and phenolic acids were the most abundant classes in terms of total peak area, with distribution depending on the plant raw materials obtained from *S. hispanica.* The main compound among this class for all types of extracts was rosmarinic acid which was proven to be the most abundant for antioxidant potential. All tested extracts exhibited considerable antibacterial and antifungal activity. The strongest bioactivity was found in leaf extracts, which presented bactericidal activity against Gram-positive bacteria (*S. aureus*, *S. epidermidis*, *M. luteus* and *E. faecalis*). The work represents the first compendium of knowledge comparing different *S. hispanica* plant raw materials in terms of the profile of biologically active metabolites and their contribution to antioxidant, antimicrobial and antifungal activity.

## 1. Introduction

Thanks to the development of advanced analytical technologies, it is now possible to better characterize the complex composition of many plant raw materials, with a focus on understanding their biologically active compounds. In this context, a very interesting plant species commonly used in phytotherapy as well as in health nutrition is chia—*Salvia hispanica* L. (Lamiaceae) [1,2]. Currently, *S. hispanica* can be field cultivated or cultivated in greenhouse conditions in many regions [3,4,5]. However, *S. hispanica* is a neglected and underutilized plant (NUCS) [6]. The plant is now known for its health-promoting properties [6,7,8]. Furthermore, chia seeds are well known in Traditional Chinese Medicine (TCM) and were used by the Mayan and Aztec tribes as food and an ingredient in many herbal mixtures. However, no specific healing properties were attributed to them at the time [1,9].

Currently, the main raw material obtained from *S. hispanica* is chia seed (*Salviae hispanicae semen*) [10]. The chemical composition and biological activities of chia seeds have been well described in the scientific literature, and therefore, commercial interest in this raw material continues to increase [11,12,13,14]. In 2009, the European Food Safety Authority (EFSA) issued a positive opinion on chia seeds, making them safe for use in the food industry [15]. In Canada, the seeds and chia seed oil are classified as “natural health product ingredient” [16]. However, few scientific studies deal with the analysis and commercial use of other raw materials derived from this plant, namely sprouts, leaves, flowers, and whole herbs.

The valuable chemical composition of chia seeds is crucial for their popular use in nutritional therapy for many diseases in our civilization. Chia seeds are valued in food production due to their high content of essential fatty acids such as α-linolenic acid and linoleic acid. In addition, the seeds are an essential source of plant protein and contain all essential amino acids (arginine, leucine, phenylalanine, lysine, valine, isoleucine, threonine, methionine, histidine and tryptophan). Chia seeds also contain high levels of dietary fiber, predominantly found in the water-insoluble fraction, as well as essential macro- and micronutrients [17,18]. Equally important is the content of biologically active compounds and antioxidants, which are responsible for the health-promoting potential of the seeds. Phenolic acids (gallic acid, ferulic acid, *p*-coumaric acid, caffeic acid), depsides (rosmarinic acid and chlorogenic acid), flavonols (kaempferol, quercetin, myricetin and rutoside), flavones (apigenin), isoflavones (daidzein, glycitin, genistein and glycitein) and flavan-3-ols (catechin, epicatechin) are present in predominant amounts [17,18,19,20]. Particularly noteworthy in the studies is the high content of rosmarinic acid, which has a broad spectrum of biological activity, including, in particular, antioxidant, anti-inflammatory and antibacterial activities [17,21,22]. Scientific studies conducted on in vitro cultures on human and animal models demonstrate the antioxidant [11,19,23,24,25,26], antidiabetic [27,28,29,30,31], hypotensive [13,30,32,33,34], hypolipemic [28,35] and hepatoprotective effects of chia seeds.

On the other hand, interestingly, there are few papers dealing with phytochemical or biological studies of the other raw materials extracted from *S. hispanica*. Only some researchers focused on the sprouts and leaves of *S. hispanica* and proved that they could be an interesting plant resource [36,37,38,39,40,41,42]. It is known that chia leaves are a source of hydroxycinnamic acid and its derivatives, flavones (apigenin, luteolin, orientin, and vitexin), and flavonols (quercetin, kaempferol derivatives). In addition, studies on chia sprouts have shown that they are a source of proteins, minerals (especially calcium and magnesium), and vitamins (especially A, E, C, and the B group) [41,43]. In addition, sprouts contain a high concentration of unspecified plant polyphenols with strong antioxidant potential [44,45]. However, there are no scientific studies detailing and comparing the phytochemical profile and antioxidant and antimicrobial activity of chia flowers or herb.

The current scientific literature on the different morphological parts of *S. hispanica* is very limited. Studies have comprehensively described the composition and biological activity of chia seeds, but knowledge of the sprouts, flowers, leaves, or herb has not been exhaustively described. Current research on the analysis of the leaves is only related to the chemical analysis of the essential oil and the content of some biologically active compounds. Few studies describe the antioxidant capacity of chia leaf extracts.

The work aims to explore the scientific knowledge on the content of the main metabolites, focusing on the group of polyphenols present in different raw materials extracted from *S. hispanica*, as well as on their antioxidant, antimicrobial and antifungal activities. The work represents a comprehensive comparative analysis of *S. hispanica* plant material—seeds, sprouts, leaves, flowers, roots and herb. The study included phytochemical and qualitative analysis by the HPLC technique in conjunction with post-column derivatization and untargeted metabolomic analysis. Quantitative analysis of the content of the predominant phenolic compound—rosmarinic acid—was performed using HPLC-DAD. The study of antioxidant activity with the indication of the main compounds responsible for this activity was performed by post-column derivatization with ABTS reagent. The study compared the antioxidant potential of the tested extracts from different morphological parts of *S. hispanica*. In addition, a comparative study of antibacterial and antifungal activity was also carried out using the microdilution broth method.

## 2. Results

### 2.1. Metabolomic Profiling Using LC-Q-Orbitrap HRMS

**Figure 1:**
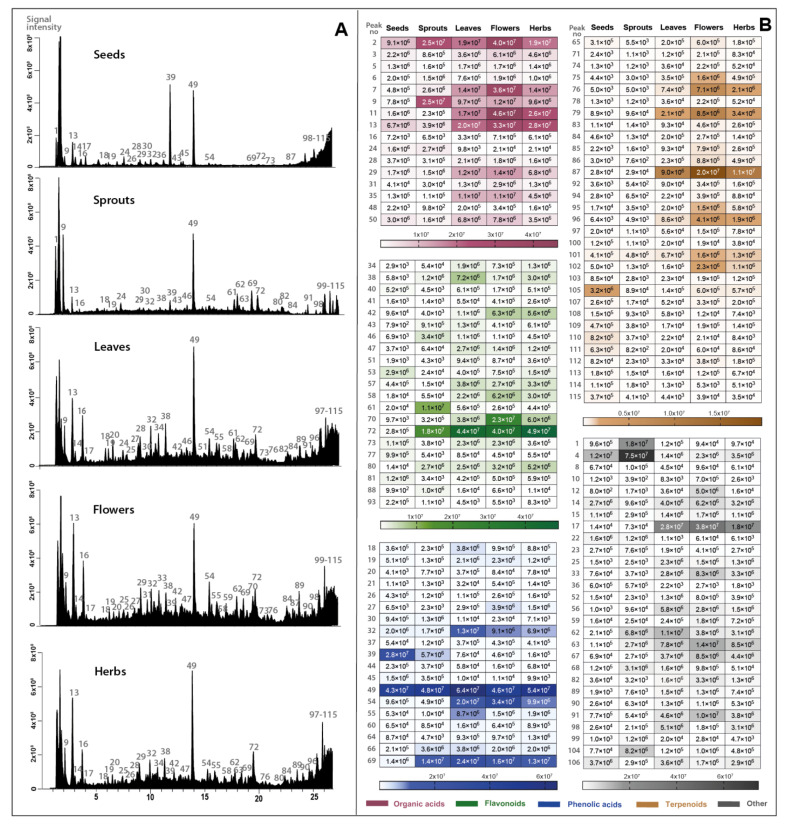
Total ion chromatograms (registered in negative-ion mode) (**A**) set with heat map representing the mean peak area value of the identified compounds (**B**) detected in extracts from different parts of *S. hispanica*. For identity of peaks, see Table 1.

The high-resolution, accurate mass via Orbitrap used in this study yielded the identification and preliminary characterization of one hundred fifteen compounds (Table 1). In Figure 1, the metabolite profiles as total ion chromatograms and heat maps with the signal intensity of individual analytes are reported. The largest class of compounds was terpenoids, with 31 compounds, followed by flavonoids (21 compounds), phenolic acids and derivatives (19 compounds), organic acids (16 compounds) and others such as fatty acids, sugars and unidentified compounds. The organic and phenolic acids were the most abundant classes in terms of total peak area with distribution depending on the type of part *S. hispanica* (Figure 1A).

This class was represented mainly by hydroxycinnamic acids and their derivatives. The main compound among this class for all types of extracts was rosmarinic acid (peak **49**) with pseudo-molecular ions at m/z 359.0769 (C_21_H_17_O_11_^−^) and fragmentation ions at *m*/*z* 197.0450 and *m*/*z* 161.0235 formed by cleavage of a caffeic acid and danshensu moieties. The deprotonated form of caffeic acid was also detected in compound **69**, which was identified as salvianolic acid F ([M-H]¯ m/z 313.07142). The extracts obtained from leaves, flowers and herbs were additionally characterized by a high content of ferulic acid (peak **54**) and caffeic acid (peak **32**) with [M-H] ^−^ ions at m/z 193.0496 and m/z 179.0336, respectively. In the case of ferulic acid, the fragmentation ion at m/z 134.0379 formed after the loss of carbon dioxide, and the methyl radical was observed. The caffeic acid precursor ion also generated characteristic major fragments at m/z 135.0441 due to the loss of carbon dioxide. Seed and sprout extracts, on the other hand, were characterized by a high content of salviaflaside (peak **39**) with pseudo-molecular ions at m/z 521.1298 (C_24_H_25_O_13_^−^). The parent ion of this rosmarinic acid glucoside produced fragment ions at m/z 359.0748 and m/z 161.0235, expected for rosmarinic acid and caffeic acid, respectively.

The highest content of organic acids was observed for flower extracts. The major compounds were assigned as gluconic (peak **2**), tartaric (peak **7**), malic (peak **9**), citric (peak **11**) and isocitric acid (**13**).

Another class of phytochemicals detected in S. *hispanica* extracts was flavonoids. Most of the identified compounds belonging to this class have been assigned to flavones. In almost all extracts, the content of flavone with peak number **72** was the highest. Compound **72** gave the precursor ion [M-H]^−^ at m/z 329.0663, indicating that its molecular formula was C_17_H_14_O_7_. It produced prominent fragment ions at m/z 299.0198 attributable to the loss of two methyl groups and m/z 271.0246 due to the further elimination of carbon monoxide. Therefore, this peak was identified as jaceosidin. Compound **70** yielded the base peak [M-H]^−^ at m/z 269.04522. Precursor and product ions at m/z 117.0333 and 151.0027 confirmed that this compound is apigenin. The glucoside, rutinoside and glucuronide of apigenin were identified by the pseudomolecular peak ions at m/z 431.0981 (peak **38**), 577.1561 (peak **42**) and 445.07747 (peak **46**), respectively, and aglycon ion in MS2 spectra formed after loss of glucoside (−162 amu), rutinoside (−308 amu) and glucuronide (−176 amu) moieties. Peaks **34**, **41** and **57** were identified as luteolin glucoside, luteolin rutinoside and luteolin, respectively, based on the presence of the ion at m/z 285.0401 in MS2 or MS spectra. Compounds **40**, **58** and **80** were identified as scutellarin, luteone glucoside and hispidulin, respectively. Compound **61,** with the highest content in sprout extracts, gave a [M−H]^−^ ion at *m/z* 345.06136 (C_17_H_13_O_8_^−^). The main fragment ion at m/z 315.0149 was attributable to the loss of two methyl groups. This compound was identified as hydroxyflavan–spinacetin.

Another main group of phytochemicals present in *S. hispanica* extracts were terpenoids. The highest content of these compounds was found in flower extracts. The most abundant compound (peak **87**) with quasimolecular ion at m/z 345.17046 (C_20_H_25_O_5_^−^) has a unique fragmentation pattern with fragmentation ions at m/z 331.1508 and 315.1597 that have been previously observed for rosmadial and hydroxyrosmadial [46]. Therefore, this compound was assigned as a rosmadial derivative. Peaks **85** and **96** with a [M−H]^−^ ions at m/z 343.15480 were assigned to isomers of rosmadial (C_20_H_23_O_5_^−^). Their parent ion generated characteristic fragments at m/z 315.1601 and m/z 299.1653 via the loss of ethylene and carbon dioxide, respectively.

In the extracts studied, especially those from sprouts, a high content of saccharides was also noted. Peaks **1** and **4** were tentatively identified as raffinose and sucrose, as they are often major transport sugars in salvia species [47]. In the case of extracts from leaves, flowers and herbs, the high content of compound **17** was also observed. Its precursor ion [M-H]^−^ was found at m/z 271.08193, which indicates that its molecular formula is C_12_H_15_O_7_^−^. This compound was tentatively identified as arbutin. The presence of fatty acids was also observed in *S. hispanica* extracts. The two with the highest concentration are compounds **62** and **91**, identified as trihydroxyoctadecadienoic acid (C_18_H_31_O_5_^−^) and dihydroxyoctadecadienoic acid (C_18_H_31_O_4_^−^), respectively.

### 2.2. Antioxidant Profiling by Post-Column Derivatization with ABTS

**Figure 2:**
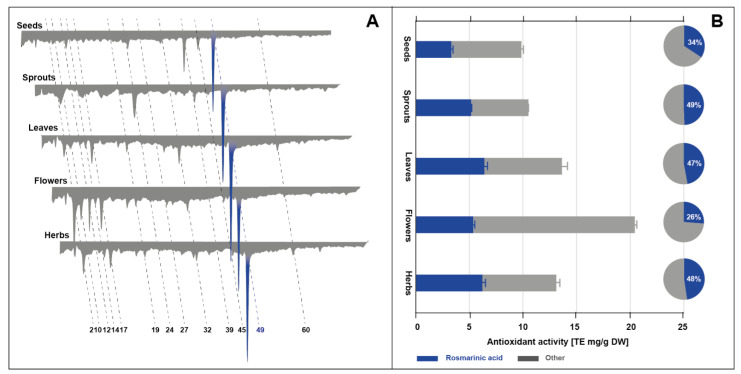
HPLC antioxidant profiles of seed, sprout, leaf, flower and herb extracts of *S. hispanica* registered at 734 nm after post-column derivatization with ABTS reagent (**A**) set with bar and pie charts reporting, respectively, antioxidant activity expressed as Trolox equivalents and the percentage contribution to the total antioxidant activity of rosmarinic acid and other antioxidants present in extracts (**B**). For identity of peaks, see Table 1.

Post-column derivatization of analytes with ABTS reagent was performed during HPLC analysis of extracts from different plant parts of *S. hispanica*. In the applied post-column derivatization, the principle action of the ABTS reagent is the same as in the case of spectrophotometric tests. The reduction reaction of the ABTS reagent leads to a significant shift in the visible UV spectrum, which results in a change in the absorption of the ABTS reagent (discoloration). Post-column introduction of the reagent into the on-line system and the presence of antioxidants in the eluate result in negative peaks in the chromatogram recorded at 734 nm (Figure 2A). The profiles obtained after derivatization indicated that several compounds identified in *S. hispanica* extracts exhibit antioxidant activity. The greatest contribution to the overall antioxidant activity was made by rosmarinic acid (peak **49**). Its activity covered 26 to 49% of the total antioxidant activity (Figure 2B). In the case of seed extract, additionally, other derivatives of hydroxycinnamic acid, such as salviaflaside (peak **39**) and dehydroxyl-rosmarinic acid-glucoside (**45**), showed antiradical potential visible as negative peaks on profile. In extracts from leaves, flowers and herbs, the presence of caffeic acid (peak **32**) also caused the reduction of the ABTS radical. Other phytochemicals showing visible antioxidant activity were gluconic acid (peak **2**), arbutin (peak **17**), danshensu (peak **19**), caftaric acid (peak **24**) and chlorogenic acid (peak **27**). The flower extract was characterized by a slightly different antioxidant profile compared to the rest of the extracts. In this case, additional antioxidants with a short retention time were noted (peaks **10**, **12**, **14**). They were probably derivatives belonging to the group of pyrimidines and purines.

Recalculation of the area of negative peaks using the calibration curve of the standard antioxidant enabled the quantification of the total antioxidant activity of the extracts in an on-line system and its expression as Trolox equivalents (Figure 2B). The highest antioxidant activity was found in flower extracts. Lower activity by about 40% and 50% was shown by extracts from leaves and herbs, as well as extracts from seeds and sprouts, respectively.

### 2.3. Analysis of the Average Content of Rosmarinic Acid Performed Using DAD-UHPLC in Extracts from Seed, Sprout, Leaf, Flower and Herb of S. hispanica

Analysis of the average content of rosmarinic acid in extracts from seeds, sprouts, leaves, flowers and herb of *S. hispanica* showed that all analyzed morphological parts have a high content of rosmarinic acid. Among all the analyzed parts of *S. hispanica*, the average content of rosmarinic acid was the highest in the leaves (198.53 mg/100 g DW). There was slightly less rosmarinic acid in the herb (185.12 mg/100 g DW). Rosmarinic acid was present in lower amounts in the flowers (149.45 mg/100 g DW), in the sprouts (134.27 mg/100 g DW) and in the least amount in the seeds (127.25 mg/100 g DW) (Table 2).

### 2.4. Antibacterial and Antifungal Activities

The antibacterial and antifungal activities of the tested extracts are presented as the MICs, i.e., the lowest concentration of compound that inhibits visible growth of the microorganism and the MBCs, i.e., the lowest concentration that results in a ≥99.9% reduction of the microorganism inoculum upon subculture to a compound-free medium (Table 3). Vancomycin, ciprofloxacin and nystatin were used as the standard drugs. Tested extracts were more active against Gram-positive reference strains. Gram-negative bacteria tested showed 4–8 times higher MIC values in comparison to those for Gram-positive bacteria. The best bioactivity was indicated for leaves extract, which presented considerable bactericidal activity against Gram-positive bacteria (*S. aureus*, *S. epidermidis*, *M. luteus* and *E. faecalis*) counted by MBC/MIC index, which equals 1–4. A stronger inhibitory effect against Gram-negative reference strains was also presented by leaves extract. The leaves extract showed the best antimicrobial activity against two Gram-positive bacteria (*S. epidermidis* and *S. aureus*). More favorable antifungal activity of chia leaves was found for *S. albicans* (MIC = 5). The whole seeds extract exhibited greater activity against *S. aureus*, *M. luteus* and *B. cereus* compared to the ground seeds extract. However, ground seeds extract demonstrated greater activity against *E. faecalis*. The sprout extract showed the best effect and the lowest MIC against two Gram-positive bacteria (*M. luteus* and *B. cereus*). The results showed that the sprout extract exhibited the best antifungal activity against *C. parapsilosis* (MIC = 0.625.). The roots extract demonstrated the best antibacterial activity against *M. luteus.* Roots extract exhibited beneficial antifungal properties against *C. parapsilosis* and *C. glabrata*. The herb extract showed the least favorable antibacterial and antifungal activity. The antibacterial efficiency of tested extracts was in the order of leaves > sprouts > whole seeds > ground seeds > roots > herb. However, their activity was much lower compared to standard drugs routinely used in bacterial infection treatment. Not strong, but still better antifungal activity against *Candida* spp. reference strains were shown for sprout extract.

## 3. Discussion

The conducted research is the first comparative analysis providing phytochemical profiling and connected with its antioxidant potential as well as antimicrobial properties of different raw materials obtained from *S. hispanica*. Rosmarinic acid was identified as the major compound responsible for antioxidant activity. The comparison of the quantity of this compound in relation to the ABTS reagent was shown. Furthermore, except for the antioxidant potential, the antimicrobial and antifungal properties were profiled for the first time while studying different *S. hispanica* raw materials. The chemical characterization revealed the presence of various groups of compounds in the extract from seeds, sprouts, leaves, flowers, and herb, mainly terpenoids (31 compounds), flavonoids (21 compounds), phenolic acids and derivatives (19 compounds), organic acids (16 compounds) and others (fatty acids, sugars, unidentified compounds).

Chia seeds are the most recognized raw material obtained from *S. hispanica*, although they are not well phytochemically profiled. A few studies described the polyphenolic profile of chia seeds, but our results are more comprehensive. Rahman et al. [48] determined the polyphenolic profile and biological activity of chia methanolic seed extract. They identified only the total phenolic content using the out-of-date method of the Singleton and Rossi assay (1965). They indicated rosmarinic acid, protocatechuic acid, *p*-hydroxybenzoic acid, *p*-coumaric acid, caffeic acid, and quercetin as the major components using HPLC-DAD-MS/MS method. The results of Oliveira-Alves et al. [49] in identifying the main phenolic compounds in methanolic chia seed extracts by LC-DAD-ESI-MS/MS methods mostly correspond with compounds identified in our study, too. The researchers confirmed the presence of phenolic acids (protocatechuic acid, *p*-hydroxybenzoic acid, cis-*p*-coumaric, cis and trans-caffeic acids, hydroxycoumaric acid, cis- and trans-ferulic acids, ellagic acid, rosmarinic acid), flavonoids (quercetin, quercetin-hexoside, kaempferol-hexoside, myricetin, apigenin, daidzein, rutin, genistein), and procyanidins (procyanidin dimer B 1, 2 and 3, procyanidin dimer A). Our results were consistent with those of Martinez-Cruz et al. [23] on 70% methanol extracts with the UHPLC method in chia seeds. The researchers indicate the main compounds as rosmarinic acid, protocatechuic acid, caffeic acid, gallic acid and daidzein. The estimated amount of rosmarinic acid was 92.67 mg/100 g DW, 1.4 times lower than the amount obtained in our results for seeds. The antioxidant activity determined by the DPPH assay indicated the high antioxidant capacity (percentage of inhibition = 68.83%) of chia seeds, which was 2 times higher than estimated in our study. The presence of phenolic acids, especially rosmarinic acid, and other phenolic compounds from isoflavones and anthocyanins were supposed to be responsible for this activity. The results were also most similar to those of Abdel-Aty et al. [50], who determined polyphenols profile using HPLC analysis. They identified in chia seed extracts: phenolic acids (gallic acid, protocatechuic acid, *p*-hydroxybenzoic acid, chlorogenic acid, caffeic acid, syringic acid, ferulic acid, synapic acid, rosmarinic acid, and cinammic acid) and flavonoids (quercetin, apigenin, chrysin). The researchers also proved that the most abundant phenolic acid identified in seeds extract was rosmarinic acid (0.320 mg/g DW). Dib et al. [51] quantified the groups of phenolic compounds in the hydromethanol extract of chia seeds. It was proved that chia seeds had a high content of total phenols (19.06 mg GAE/g DW), which were mainly represented by flavonoids (12.3 mg CE/g DW) and tannins (8.32 mg catechin equivalents (CE)/g DW). Moreover, the researchers assessed the antioxidant properties of the studied extract using DPPH and FRAP assay. The results indicated that the extract showed the highest DPPH scavenging potential with an IC50 of 0.27 mg/mL and FRAP assay with an EC50 of 0.06 mg/mL. In our study, qualitative and quantitative analysis of the main polyphenolic compounds present in chia seed extract demonstrated that the quantitatively dominant phenolic compound responsible for antioxidant activity was rosmarinic acid. The antioxidant activity of chia seeds has also been confirmed by other scientists, who have shown that chia seed extracts were able to scavenge DPPH radicals [52,53,54,55,56]. Tepe et al. [57] examined the antioxidant activity of an ethanolic extract of chia seeds and claimed that polyphenols present in chia seeds significantly inhibited oxygen free radicals. The same results were obtained by Craig et al. [58], who proved that the presence of polyphenols in chia seeds protects them from oxidative degradation. All performed studies confirm that rosmarinic acid was the main compound detected and quantified in chia seeds [23]. Similarly, the compound identified by our team—danshensu, is a simple polyphenol (3-(3,4-dihydroxyphenyl)lactic acid) corresponding to the hydrated form of caffeic acid [59]. This compound is also described in plants in the Lamiaceae family [60,61]. Our study compared the quantitative content of rosmarinic acid in *S. hispanica* seed extracts with other literature data. In addition to the present study, rosmarinic acid was also quantified in *S. hispanica* seed extracts at two other centers. In the present experiment, the content of rosmarinic acid in the seed extracts was 34.98 mg/100 g DW. which was almost 2 times less than the content determined by Pellegrini et al. [62] and 2.65 times less than the content estimated by Martinez-Cruz et al. [23]. Moreover, our study also examined the antioxidant properties of chia seed extracts and determined the percentage effect of rosmarinic acid content on this activity (which was indicated at 34.33%). We proved that other derivatives of hydroxycinnamic acids, such as salviaflaside and dehydroxyl-rosmarinic acid-glucoside, also exhibited antiradical visible potential.

Chia seeds are the main raw material obtained from *S. hispanica*, but according to current scientific studies, compared to seeds, chia sprouts could have better nutritional value and antioxidant capacity, making them a new promising plant raw material of potential medical and agri-food utilities [63,64]. Our studies showed the metabolite profile of chia sprout extracts for the first time. We identified new compounds that had never been identified by researchers before. In sprouts extract, we identified organic acids—gluconic acid, xylonic acid, threonic acid, tartaric acid, quinic acid, malic acid, citric acid, isocitric acid, homocitric acid, caftaric acid, salicylic acid, tuberonic acid hexoside, phenolic acids and their derivatives—dihydroxybenzoic acid hexoside, danshensu, neochlorogenic acid, caffeic acid, salviaflaside, rosmarinic acid, dehydroxyl-rosmarinic acid-glucoside, ferulic acid, salvianolic acid F, 4-hydroxybenzoic acid, methyl rosmarinate, feruoyl arabinose, rabdossin, caffeoyl glucose. Namely, sprout extracts were characterized by a high content of salviaflaside and jaceosidin. Moreover, compared to all extracts we analyzed, the extracts from sprouts contained high amounts of saccharides (mainly raffinose and sucrose). In our study, we proved that the main group of secondary metabolites of chia sprout extracts were derivatives of hydroxycinnamic acids. Organic acids and flavonoids occupy the next position. We have shown that the proportion (%) of rosmarinic acid in the antioxidant activity of sprout extracts determined using post-column derivatization of analytes with ABTS reagent was the highest compared to the other analyzed extracts from raw materials obtained from *S. hispanica*. The analysis showed that chia sprouts had the highest percentage content of rosmarinic acid, which contributed to antioxidant activity, at 49.5%. These results are innovative because there are no scientific studies analyzing the phytochemical profile and antioxidant properties of chia sprouts extract. Calvo-Lerma et al. [36] indicated that chia sprouts extract contains higher total polyphenol content than seeds (2.87 vs. 1.78 mg GA/g DW). Their determination of the total antioxidant activity using the DPPH assay showed higher results in sprouts in comparison to seed extracts (5.69 vs. 3.49 mg TX/g DW), which is consistent with the results obtained by our team. Abdel-Aty et al. [50] evaluated the effect of the germination process of *S. hispanica* seeds on total phenolic and flavonoid contents and antioxidant and antimicrobial properties. In the chia sprout methanolic (80%) extracts with the HPLC method, 12 phenolic acids (gallic acid, protocatechuic acid, *p*-hydroxybenzoic acid, chlorogenic acid, caffeic acid, syringic acid, vanilic acid, ferulic acid, synapic acid, *p*-coumaric acid, rosmarinic acid, and cinammic acid) and 5 flavonoids (catechin, quercetin, apigenin, kaempferol, chrysin) were identified with concentrations ranging from 0.06 to 0.80 mg/g DW. Our results presented different data. We did not detect protocatechuic acid, vanilic acid, *p*-coumaric acid and cinammic acid in the sprout extracts. The researchers showed that the dominant phenolic compound found in chia sprout extracts was protocatechuic acid (0.50 mg/g DW), followed by rosmarinic acid (0.60 mg/g DW). In our study, we proved that rosmarinic acid was the abundant compound (134.27 mg/100 g DW) identified in sprout extracts.

Previously, only a few scientific studies dealt with chia leaves. The results demonstrated the presence of fatty acids, flavonoids and essential oil. Therefore, most scientific research focuses on analyzing components isolated from chia leaf essential oil [65,66,67,68,69,70]. In our study, we showed that phenolic acids derivatives (dominant compounds: rosmarinic acid, ferulic acid, caffeic acid, 4-hydroxybenzoic acid), flavonoids (dominant compounds: vitexin, jaceosidin) and organic acids (dominant compounds: gluconic acid, tartaric acid, malic acid, citric acid, isocitric acid) were predominant in studied *S. hispanica* leaves methanolic extract.

Among phenolic acids, the most abundant in leaves extract was rosmarinic acid. These results are in line with those obtained by Amato et al. [40], who analyzed the methanolic extracts of chia leaf using the HPLC-ESI-MS method. In the study conducted by us in the chia plant material, we identified hydroxycinnamic acids and their derivatives, especially flavonoids, mainly flavones, such as apigenin, luteolin, orientin, vitexin, jaceosidin and phenolic acids with dominant amounts of rosmarinic, ferulic, isocitric, caffeic acid and their derivatives. These compounds have been commonly found in other members of the genus *Salvia* before [71,72]. Similar results were obtained by Zúñiga-López et al. [73], who identified the phenolic composition of chia leaves using the UHPLC-HRMS method. However, these researchers identified only 18 bioactive compounds in the chia leaf extracts: organic acids (dominant compounds: protocatechuic acid, *p*-coumaric acid, quinic acid, sinapic acid), phenolic acids derivatives (dominant compounds: chlorogenic acid, rosmarinic acid, caffeic acid, ferulic acid) flavonoids (dominant compounds: orientin, acetyl orientin, vitexin, coumaroyl, luteolin-O-glucuronide, kaempferol, genistein, naringenin, salvianolic acid F isomer, and dimethyl quercetin). Most of the detected compounds overlap with those identified in our study, but in our study, we identified 115 compounds (Table 1). There are very limited studies on the correlation between metabolomic analyses and the antioxidant properties of chia leaves. In our study, analysis of antioxidant activity determined by post-column derivatization with ABTS reagent of the chia leaf extracts showed that rosmarinic acid present in the leaf extracts was the most abundant compound responsible for this activity. Amato et al. [40], for potential antioxidant measurements of *S. hispanica* leaf extracts, used another three assays: oxygen radical absorbance capacity (ORAC), ORAC-Fluorescein (ORAC-FLORAC-FL index values) and 2,2′-diphenyl-1-picrylhydrazyl (DPPH). They proved that the methanolic extract of chia leaves exhibited higher antioxidant activity and indicated that rosmarinic acid was the most reactive compound, which is equivalent to the results obtained in our study. In our study, we broadened the results, and we pointed out that not only was rosmarinic acid responsible for antioxidant potential, but also other phytochemicals, such as caffeic acid, gluconic acid, arbutin, danshensu, caftaric acid and chlorogenic acid. Moreover, for the first time, we performed a recalculation of the area of negative peaks using the calibration curve of the antioxidant standard, which allowed us to quantify the total antioxidant activity of the leaf extracts in the on-line system. As a result, quantification of the total antioxidant activity of the extract expressed as Trolox equivalents and the percentage of the total antioxidant activity of the rosmarinic acids and other antioxidants present in the extracts showed that the antioxidant activity for the leaf extract was about 40–50%.

The study conducted by our team for the first time assessed the main metabolites and evaluated the antioxidant activity of *S. hispanica* flower extracts. The main group of secondary metabolites found in the flower extracts were organic acids (dominant compounds: gluconic acid, tartaric acid, citric acid, isocitric acid), flavonoids (dominant compounds: apigenin, jaceosidin), hydroxycinnamic acids and their derivatives (dominant compounds: caffeic acid, rosmarinic acid, ferulic acid, salvianolic acid F), and terpenoids (dominant compounds: hydroxyrosmadial, carnosic acid isomer, rosmadial derivative, rosmadial isomer, rosmanol, carnosol isomer). We showed that the main compound identified in flower extracts was rosmarinic acid. The maximum content of rosmarinic acid was determined in the flower extracts and was equal to 369.09 mg/100 g DW. Comparative analysis of the percentage contribution of rosmarinic acid to the antioxidant activity of all tested extracts from *S. hispanica* raw materials showed that the flower extracts contribute the least to antioxidant activity—26.3%. In our study, flower extracts demonstrated a slightly different antioxidant profile compared to the rest of the analyzed extracts. Probably the presence of derivatives belonging to the group of pyrimidines and purines was responsible for this activity.

Our study proved that the *S. hispanica* herb metabolite profile was the most abundant in organic acids (dominant compounds: citric acid, isocitric acid), phenolic acids (dominant compounds: rosmarinic acid, ferulic acid, salvianolic acid F) followed by flavonoids (dominant compounds: apigenin rutinoside, apigenin, jaceosidin, hispidulin), terpenoids and saccharides (dominant compounds: hydroxyrosmadial, carnosic acid isomer, rosmadial derivative, rosmadial isomer). The quantitively dominant compound for herb was also rosmarinic acid. Formerly, only Dziadek et al. [42] investigated the phytochemical profile of *S. hispanica* herb extracts. They identified the polyphenol profile by HPLC analysis. They found in herb extracts only the following compounds: *p*-hydroxybenzoic acid, caffeic acid, chlorogenic acid, ferulic acid, gallic acid, *p*-coumaric acid, rosmarinic acid, synapic acid, syringic acid, vanillic acid, acacetin, apigenin, catechin, epicatechin, hesperidin, hispidulin, isorhamnetin, kaempferol, luteolin, myricetin, naringin, quercetin, rutin, carnosic acid, and carnosol. In our study, a greater number of compounds were identified. We did not confirm the presence of chlorogenic acid, gallic acid, *p*-coumaric acid, synapic acid, syringic acid, vanillic acid, catechin, epicatechin, isorhamnetin, myricetin, naringin, quercetin and rutin. Dziadek et al. [42] also determined the antioxidant power of *S. hispanica* herb extracts at 716.26 μmol TX/g DW. In our study, we demonstrated that rosmarinic acid contributes to this activity. The percentage contribution of rosmarinic acid to the antioxidant activity of herb extracts was 47.66% (185.12 mg/100 g DW). Abou Zeid et al. [74] studied the aerial parts of *S. hispanica* by the UPLC-ESI-MS/MS technique. They identified significantly fewer compounds than in our study (37 compounds) from phenolic acids, flavonoids, tannins, diterpenoids, lignans and triterpenoids. The individual compounds: caffeic acid, rosmarinic acid, ferulic acid, orientin, vitexin, danshensu, carnosol, jaceosidin, syringetin and luteolin, are similar to these confirmed in the present study. Furthermore, Abou Zeid et al. performed the analysis of antioxidant properties by the DDPH method and demonstrated the significant potential of the ethyl acetate extracts of *S. hispanica* aerial parts (herb).

There is limited research on the antibacterial and antifungal actives of *S. hispanica* seeds, sprouts, leaves, roots and herb extract. Our study showed that chia leaf extracts exhibited the highest antibacterial and antifungal activity compared to the other tested extracts. Chia leaf extracts showed stronger antibacterial activity against two Gram-positive bacteria (*S. epidermidis* and *S. aureus*). More favorable antifungal activity of chia leaves was found for *S. albicans*. The sprout extracts demonstrated the best effect and lowest MIC against two Gram-positive bacteria (*M. luteus* and *B. cereus*). The results proved that the sprout extracts showed the best antifungal activity against *C. parapsilosis*. The herb extracts showed the least favorable antibacterial and antifungal activity. The antibacterial efficacy of the tested extracts was in the order of leaves > sprouts > herb. However, their activity was significantly lower compared to that of standard drugs routinely used to treat bacterial infections. Abdel-Aty et al. [50] examined the antimicrobial activity of chia sprouts raw chia seed extracts against Gram-negative bacteria (*E. coli* O157-H7 ATCC 51,659, *Salmonella typhi* ATCC 15,566 and *Pseudomonas aeruginosa* NRRL B-272) and one Gram-positive bacterium (*Staphylococcus aureus* ATCC 13,565). The range of MIC for the chia sprout extracts was lower (0.40–0.65 mg/mL) in comparison to the dry chia seed extracts. In our study, the activity of the chia sprout extracts was a bit less (the MIC range value 0.625–10 mg/mL). The antimicrobial activity of chia protein hydrolysates obtained from seeds was studied by Coelho et al. [75]. The protein hydrolysates exhibited favorable inhibitory activity against *S. aureus* to a greater extent compared to *E. coli*, which is in line with our results confirming the more favorable antimicrobial activity of all analyzed extracts against *S. aureus* compared to *E. coli*. Güzel et al. [76] investigated the antibacterial and antifungal activity of ethanol extract of chia seeds against reference strains of *S. aureus*, *B. subtilis*, *E. coli*, *A. baumannii*, *A. hydrophila*, *C. albicans*, *C. tropicalis* and *C. glabrata* showing their higher activity compared to those presented in this study. According to the recent literature, the composition and content of key bioactive compounds in chia seeds can vary depending on external factors such as geographic origin, climatic conditions, agricultural practices, extraction procedures and antimicrobial activity procedures [9,77]. These factors may affect the efficacy of the extract under study and may result in different outcomes compared to other studies. In addition, Güzel et al. demonstrated that chia seed extract exhibited the highest antifungal activity against *C. glabrata*, but the result was not as high as with fluconazole (MIC values: 31.25 µg/mL and 3.90 µg/mL, respectively). In our study, we found significantly higher antifungal activity of the whole seed extract compared to the ground seed extract. The antimicrobial effect of chia seeds is likely due to their rich chemical composition. Chia seeds are a source of fatty acids, accounting for about 30%, which include linoleic acid (17–26%) and linolenic acid (50–57%). Chia seeds are also a source of vitamins, macronutrients and micronutrients [9,78]. The presence of numerous antioxidants in chia seeds, such as omega-3 fatty acids, may determine their antimicrobial properties. Chia seeds contain kaempferol and quercetin, which have scientifically proven antibacterial properties. It can be inferred that kaempferol binds to an enzyme in bacterial cells and blocks a process essential for bacterial function. Chia seeds also contain caffeic acid and *p*-coumaric acid, which have proven antimicrobial activity [79,80]. Moreover, adding chia seeds to food products can increase their microbiological stability and prevent contamination without additional preservatives. Numerous human pathogens have been scientifically proven to be experimentally sensitive to the inhibitory effects of phenolic acids, flavonoids, tannins and anthocyanins, especially against several specific strains of Gram-positive bacteria (*S. aureus*, *L. monocytogenes*, *M. luteus*, *E. faecalis*, *C. botulinum* and *B. subtilis*) and Gram-negative bacteria (*E. coli*, *S. typhimurium*, *S. enterica*, *P. mirabilis*, *Y. enterocolityca*, *S. dysenteriae*, *S. flexneri*, *P. fluorescens*, *P. aeruginosa* and *V. cholerae*) and the fungal pathogen *C. albicans* [69,76,79,81,82,83,84,85,86].

In our study, we examined for the first time the antibacterial and antifungal properties of the root extract of the *S. hispanica* plant. The roots extract demonstrated beneficial antifungal properties against *C. parapsilosis* and *C. glabrata*.

## 4. Materials and Methods

### 4.1. Materials and Chemicals

Reagents of analytical, HPLC or MS grade, including acetonitrile, methanol, water, and formic acid, reagents for antioxidant profiling: 2,2′-azinobis(3 ethylbenzothiazoline-6-sulfonic acid) diammonium salt (ABTS) and (±)-6-hydroxy-2,5,7,8-tetramethylchromane-2-carboxylic acid (Trolox) and rosmarinic acid standard were purchased from Sigma- Aldrich (St. Louis, MO, USA. Reference strains came from American Type Culture Collection (ATCC) (LGC Standards, Teddington, UK), Mueller–Hinton broth and agar were purchased from Oxoid Ltd. (Hampshire England); glucose, dimethyl sulfoxide (DMSO) were purchased from Avantor Performance Materials Poland S.A. (Gliwice, Poland); sterile physiological saline (BioMerieux, Craponne, France).

### 4.2. Plant Material

The seeds from which the plant material for analysis was obtained were from Guatemala, obtained from KruKam Polska S.A. (Wodzisław Śląski, Poland). The cultivation of *S. hispanica* was carried out under greenhouse conditions in the Prof. Marian Koczwara Medicinal Plants Garden of the Faculty of Pharmacy of the Jagiellonian University Medical College (Cracow, Poland). The herb, leaves and flowers were harvested in August 2021 during the flowering and fruiting period of the plants. Sprout culture was performed in a PlantiCo brand germinator (Stare Babice, Poland). The sprouts were collected in August 2021. The plant material was dried by freeze-drying (Labconco freeze-dryer, Kanas City, MO, USA).

### 4.3. Preparation of Extracts

For chromatographic analysis, the powdered lyophilizates (50 mg) were extracted with a methanol solution (70%, 0.5 mL). The extraction was assisted by ultrasound (15 min). The extracts were centrifuged (13,000 rpm, 15 min), and the supernatants were collected. The extraction step was repeated for the solid residue with another portion of methanol (70%, 0.5 mL). The combined supernatants (~1 mL) were subjected to chromatographic analysis.

To prepare the extracts used for the analysis of antibacterial and antifungal properties, samples of dry powdered plant tissue were weighed at 4 g DW each. The material was extracted with methanol in a volume of 100 mL. Extraction was carried out in an ultrasonic bath model 3 times for 20 min each. The extracts were filtered through tissue paper strainers. The material was extracted using blotting paper, which was poured into crystallizers after draining. The material was left to evaporate for 3 days. After 3 days, the material was eluted with methanol, and the weighed extract was placed in 7 mL (16 × 66 mm) polypropylene tubes from Rymed Company (Dabrowa Gornicza, Poland).

### 4.4. Untargeted Metabolomics by LC-Q-Orbitrap HRMS

The *Salvia hispanica* hydromethanolic extracts were investigated using a Dionex Ultimate 3000 UHPLC system (Thermo ScientificTM, Dionex, San Jose, CA, USA). Chromatography separations were performed using SynergiTM Hydro-RP A (150 × 4.5 mm, 4 µm, Phenomenex) column. Mobile phases A (water) and B (acetonitrile), both acidified with formic acid (0.1% *v*/*v*), were pumped at a flow rate of 0.8 mL/min^1^, according to the following gradient pattern: 0 min, 5% B; 20 min, 50% B; 25 min, 100% B; 27 min, 100% B and finally, the initial conditions were held for 8 min as a re-equilibration step. The injection volume was 4 μL. The chromatographic unit was coupled to a Q ExactiveTM Focus quadrupole-Orbitrap mass spectrometer (Thermo Fisher Scientific, Bremen, Germany) with a heated electrospray ionization source (HESI II). The HESI parameters in negative polarity included: sheath gas flow rate, 35 arb; auxiliary gas flow rate, 15 arb; sweep gas flow rate, 3 arb; spray voltage, 2.5 kV; capillary temperature, 350 °C; S-lens RF level, 50; heater temperature, 300 °C. Full scan data in the negative mode was acquired at a resolving power of 70,000 FWHM; AGC target, 1e6; max IT, auto. A scan range of m/z 100–1200 was chosen for the compounds of interest. The parameters of data-dependent MS2 were as follows: resolution, 17,500; isolation window, 3.0 m/z; normalized collision energy, 30; AGC target, 1e6; max IT, auto. Mass calibration was performed once a week, in both positive and negative modes, using mixture containing n-butylamine, caffeine, Met-Arg-Phe-Ala (MRFA) and Ultramark 1621.

Raw data from high-resolution mass spectrometry were elaborated with Compound Discoverer (v. 2.1, Thermo, Waltham, MA, USA). Major metabolite identification was based on accurate mass and mass fragmentation pattern spectra against MS-MS spectra of compounds available on customized database of different classes of phytochemicals created on the basis of literature data on the *Salvia* species and implemented in the software. Raw data from three experimental replicates and a blank sample were processed using a workflow presented in Kusznierewicz et al. [87].

### 4.5. Antioxidant Profiling by Post-Colum Derivatization with ABTS

Antioxidant profiles were obtained for *S. hispanica* hydromethanolic extracts using an HPLC-DAD system (Agilent Technologies, 1200 series, Waldbronn, Germany) coupled with a Pinnacle PCX Derivatization Instrument (Pickering Laboratories Inc.) and a UV-Vis detector (Agilent Technologies). The chromatographic column and conditions of chromatographic separation were the same as in the case of LC-HRMS analysis. The post-column derivatization with ABTS reagent was carried out according to Kusznierewicz et al. [88,89] with slight modification. Stream of methanolic ABTS solution (1 mM) was introduced to the eluate stream at a rate of 0.1 mL/min and then directed to the reaction loop (1 mL, 130 °C). Reduction of ABTS radical by extract components was monitored at 734 nm. The antioxidant activity of the major reducing analytes was quantified with the use of Trolox calibration curve and expressed as Trolox equivalents. The percentage contribution of the rosmarinic acid to the antioxidant activity of extracts was estimated on the assumption that 100% is the sum of the negative peak areas integrated into chromatograms obtained after derivatization with ABTS.

### 4.6. Rosmarinic Acid Determination

For quantitative determination of rosmarinic acid, the calibration curve was generated by integrating the areas of absorption peaks (330 nm) determined during HPLC-DAD analysis of serial dilutions of authentic standard. The chromatographic system, column and conditions of separation were the same as in the case of antioxidant profiling (Section 4.5).

### 4.7. Antibacterial and Antifungal Activities

All extracts were screened for antibacterial and antifungal activities by two-fold micro-dilution broth method. Minimal inhibitory concentration (MIC) of tested compounds were evaluated for the panel of reference Gram-positive bacteria: *Staphylococcus aureus* ATCC 25923, *S. aureus* ATCC BAA-1707, *Staphylococcus epidermidis* ATCC 12228, *Micrococcus luteus* ATCC 10240, *Bacillus cereus* ATCC 10876 and Gram-negative bacteria: *Salmonella* Typhimurium ATCC 14028, *Escherichia coli* ATCC 25922, *Proteus mirabilis* ATCC 12453, *Klebsiella pneumoniae* ATCC 13883, *Pseudomonas aeruginosa* ATCC 9027 and yeasts: *Candida albicans* ATCC 102231, *Candida parapsilosis* ATCC 22019, *Candida glabrata* ATCC 90030. The procedure for conducting antimicrobial activity testing has been described in detail before [90]. Briefly, the solutions of tested compounds dissolved in dimethylosulfoxide (DMSO) were suspended in Mueller–Hinton broth for bacteria or Mueller–Hinton broth with 2% glucose for fungi. Then the series of two-fold dilutions were carried out in the sterile 96-well polystyrene microtitrate plates (Nunc, Denmark), obtaining concentration range from 10 to 0.078 mg/mL in the appropriate medium. Simultaneously, the inocula of 24 h cultures of microorganisms in sterile physiological saline (0.5 McFarland standard density) were prepared and added to each well, obtaining final density of 5 × 10^5^ CFU/mL for bacteria and 5 × 10^4^ CFU/mL for yeasts; CFU—colony forming units. Proper positive (inoculum without tested compound) and negative (compound without inoculum) controls were added to each microplate. Vancomycin, ciprofloxacin and nystatin were used as the standard reference reagents. After incubation (35 °C, 24 h), the growth of microorganisms was measured spectrophotometrically at 600 nm (BioTEK ELx808, BioTek Instruments, Inc, Winooski, VT, USA). MICs were marked at the lowest dilution of extract without the growth of bacteria or fungi. Then, 5 µL of the suspension from each well, including controls, was subcultured on the agar plates in order to determine the minimal bactericidal concentration (MBC) or minimal fungicidal concentration (MFC). The plates were incubated at 35 °C for 24 h. The MBC/MFC was determined at the lowest concentration of extracts inhibiting the growth of microbes. MBC/MIC index was also calculated to show bacteriostatic or bactericidal effect of tested extracts.

## 5. Conclusions

In the current literature, there are no scientific studies on the comparative analysis of different plant raw materials obtained from *S. hispanica*. In addition, studies on the antioxidant, antimicrobial and antifungal activities of extracts from different chia raw materials are severely limited. Our work is innovative because it conducts an in-depth characterization and analysis comparing the antioxidant, antimicrobial and antifungal properties of all morphological parts of *S. hispanica*. In this study, for the first time, the phytochemical profiling and comparative analysis of various morphological parts/organs of *S. hispanica* extracts was conducted. We showed that in *S. hispanica* raw materials, the largest class of compounds were terpenoids, followed by flavonoids, phenolic acids and derivatives, organic acids, and other compounds, such as fatty acids and sugars. Conducted analyses proved that organic and phenolic acids were the most abundant class of phytochemicals identified in studied extracts. Rosmarinic acid, belonging to the hydroxycinnamic acids group, was the quantitatively dominant compound found in all tested extracts.

The greatest contribution to overall antioxidant activity was made by this compound. Rosmarinic acid contribution to total antioxidant activity was the highest for sprout, herb and leaf extracts (49.5, 47.7 and 47.1%, respectively) and the lowest for seed and flower extracts (34.3 and 26.3%, respectively). The contribution to the antioxidant activity of sprout, herb and leaf extracts was 144, 139 and 137 times stronger compared to seeds extract.

The results of the antibacterial and antifungal activities of the tested extracts proved their higher activity against Gram-positive than Gram-negative reference strains. In Gram-negative bacteria, the tested extracts showed 4–8 times higher MIC values compared to those for Gram-positive bacteria. The antibacterial efficiency of tested extracts was in the order of leaves > sprouts > whole seeds > ground seeds > roots > herb. However, compared to the leaf and herb extracts, the sprout extracts showed better antifungal activity against *Candida* spp. reference strains.

In conclusion, the results obtained in our study indicate that not only the seeds but also other morphological parts of *S. hispanica* may be a potential source of novel raw materials containing compounds with strong antioxidant, antimicrobial and antifungal potential.

## Figures and Tables

**Table 1:** Retention times (RT, min), proposed formulas, experimental m/z, accuracy (Δ, ppm) and main diagnostic experimental product ions of the major compounds identified by LC-Q-Orbitrap HRMS in seed, sprout, leaf, flower and herb extracts of *S. hispanica* in ESI(−).

No.	RT [min]	Name	Proposed Formula	Theoretical (m/z)	Observed (m/z)	Δ (ppm)	Fragment Ions (m/z)	Class *
**1**	1.86	Raffinose	C_25_H_28_O_11_	503.15534	503.15543	−0.2	89.0230; 71.0124; 101.0231; 59.0125; 113.0231	S
**2**	1.88	Gluconic acid	C_6_H_12_O_7_	195.05048	195.04985	3.2	75.0073; 59.0125; 72.9917; 71.0124; 87.0074	OA
**3**	1.91	Xylonic acid	C_5_H_10_O_6_	165.03992	165.03904	5.3	87.0074; 75.0073; 71.0124; 59.0125; 72.9917	OA
**4**	1.95	Sucrose	C_12_H_22_O_11_	341.10839	341.10850	−0.3	59.0125; 89.023; 71.0124; 101.023; 113.0231	S
**5**	1.95	Threonic acid	C_4_H_8_O_5_	135.02935	135.02827	8.0	75.0073; 71.0124; 72.9917; 59.0125; 55.0176	OA
**6**	2.01	Quinic acid	C_7_H_12_O_6_	191.05557	191.05491	3.4	85.0281; 93.0332; 59.0125; 87.0074; 71.0124	OA
**7**	2.02	Tartaric acid	C4H6O6	149.00862	149.00763	6.6	72.9917; 59.0125; 87.0074; 73.9951; 68.9968	OA
**8**	2.09	Heptose	C_7_H_14_O_7_	209.06613	209.06571	2.0	85.0281; 57.0332; 59.0125; 55.0176; 71.0124	S
**9**	2.29	Malic acid	C_4_H_6_O_5_	133.01370	133.01258	8.4	71.0125; 72.9917; 59.0125; 72.0158; 115.0022	OA
**10**	2.35	Uric acid isomer	C_5_H_4_N_4_O_3_	167.02052	167.02071	−1.1	69.008; 96.019; 124.014; 97.0029; 81.008	P
**11**	2.67	Citric acid	C_6_H_8_O_7_	191.01918	191.01843	3.9	87.0074; 111.0075; 57.0332; 85.0281; 67.0175	OA
**12**	2.77	Uric acid isomer	C_5_H_4_N_4_O_3_	167.02052	167.01958	5.6	69.008; 96.019; 124.0139; 97.0029; 110.9332	P
**13**	3.03	Isocitric acid	C_6_H_8_O_7_	191.01918	191.01845	3.8	87.0073; 111.0074; 57.0332; 85.0281; 67.0175	OA
**14**	3.14	Pseudouridine	C_9_H_12_N_2_O_6_	243.06171	243.06187	−0.6	82.0284; 110.0234; 66.0335; 118.9651; 146.9601	P
**15**	3.56	Methoxyguanosine	C_11_H_15_N_5_O_6_	312.09441	312.09476	−1.1	134.0461; 146.9601; 135.0507; 148.9558; 254.889	P
**16**	3.39	Homocitric acid	C_7_H_10_O_7_	205.03483	205.03441	2.1	71.0488; 101.023; 99.0438; 115.0385; 125.0231	OA
**17**	3.80	Arbutin	C_12_H_16_O_7_	271.08178	271.08193	−0.5	108.0204; 109.0235; 71.0124; 85.0281; 123.0441	H
**18**	5.87	Dihydroxybenzoic acid hexoside	C_13_H_16_O_9_	315.07161	315.07193	−1.0	108.0204; 152.0105; 109.0288; 112.9843; 68.9944	PAD
**19**	6.13	Danshensu	C_9_H_10_O_5_	197.04500	197.04456	2.2	72.9917; 123.0439; 135.0441; 134.0361; 122.0361	PAD
**20**	6.42	Dihydroxybenzoic acid hexoside	C_13_H_16_O_9_	315.07161	315.07191	−0.9	109.0282; 153.0183; 152.0105; 112.9843; 68.9943	PAD
**21**	7.00	Neochlorogenic acid	C_16_H_18_O_9_	353.08726	353.08754	−0.8	191.0554; 135.044; 179.0341; 192.0587; 136.0473	PAD
**22**	7.04	Unknown	C_75_H_57_O_12_	1148.37718	1148.37575	−1.3	1148.377; 1149.3793; 1026.3395; 1027.3422; 127.6119	-
**23**	7.44	D-(+)-Tryptophan	C_11_H_12_N_2_O_2_	203.08205	203.08161	2.2	116.0493; 74.0234; 142.0652; 117.0527; 72.0076	AA
**24**	7.48	Caftaric acid	C_13_H_12_O_9_	311.04031	311.04066	−1.1	135.044; 149.0081; 179.0341; 87.0074; 136.0473	OA
**25**	7.64	Unknown	C_20_H_36_O_11_	451.21794	451.21830	−0.8	167.1068; 89.023; 71.0124; 119.0337; 59.0125	-
**26**	8.21	Caffeoyl glucose	C_15_H_18_O_9_	341.08726	341.08739	−0.4	135.044; 179.0342; 180.0376; 136.0474; 134.0368	PAD
**27**	8.48	Chlorogenic acid	C_16_H_18_O_9_	353.08726	353.08745	−0.5	191.0554; 85.0281; 161.0234; 93.0331; 135.0438	PAD
**28**	8.87	Salicylic acid	C_7_H_6_O_3_	137.02387	137.02389	−0.1	108.0204; 136.0154; 137.0233; 91.0176; 65.0019	OA
**29**	9.15	Tuberonic acid hexoside	C_18_H_28_O_9_	387.16551	387.16584	−0.8	59.0125; 89.023; 101.0231; 71.0124; 207.102	OA
**30**	9.49	Feruloyl arabinose	C_14_H_14_O_9_	325.05596	325.05622	−0.8	134.0362; 193.05; 112.9847; 117.0334; 135.0395	PAD
**31**	9.80	Tuberonic acid hexoside	C_18_H_28_O_9_	387.16551	387.16582	−0.8	59.0125; 89.0230; 163.1119; 71.0124; 101.023	OA
**32**	9.95	Caffeic acid	C_9_H_8_O_4_	179.03444	179.03362	4.6	135.0441; 134.0362; 89.0383; 107.0491; 136.0473	PAD
**33**	10.47	Unknown	C_17_H_30_O_9_	377.18116	377.18142	−0.7	59.0125; 71.0124; 112.9844; 377.1813; 89.023	-
**34**	10.64	Orientin	C_21_H_20_O_11_	447.09274	447.09305	−0.7	327.0511; 357.0618; 328.0545; 297.0406; 285.0406	FV
**35**	10.82	Tuberonic acid	C_12_H_18_O_4_	225.11269	225.11245	1.1	59.0125; 97.0645; 68.9944; 81.0331; 95.0489	OA
**36**	10.86	Unknown	C_75_H_55_O_11_	1130.36658	1130.36512	−1.3	1131.3685; 1132.3582; 1133.3661; 239.0889; 652.0154	-
**37**	10.92	Przewalskinic acid A	C_18_H_14_O_8_	357.06105	357.06142	−1.0	109.0282; 159.0442; 269.0817; 135.0441; 175.0392	PAD
**38**	11.46	Vitexin	C_21_H_20_O_10_	431.09783	431.09814	−0.7	311.0564; 283.0613; 312.0597; 341.0668; 269.0458	FV
**39**	11.86	Salviaflaside	C_24_H_26_O_13_	521.12952	521.12978	−0.5	161.0235; 323.0773; 359.0748; 179.0341; 324.0808	PAD
**40**	12.00	Scutellarin	C_21_H_18_O_12_	461.07201	461.07254	−1.1	285.0407; 286.044; 113.0232; 85.0282; 112.9843	FV
**41**	12.23	Luteolin rutinoside	C_27_H_30_O_15_	593.15065	593.15118	−0.9	285.0403; 593.1502; 284.0327; 594.1534; 269.0456	FV
**42**	12.38	Apigenin rutinoside	C_27_H_30_O_14_	577.15574	577.15610	−0.6	269.0455; 270.0489; 577.1547; 311.0541; 112.9841	FV
**43**	12.48	Apigenin-malonyl glucoside	C_24_H_22_O_13_	517.09822	517.09854	−0.6	311.0564; 413.088; 312.0597; 341.0667; 283.0614	FV
**44**	12.75	Rabdosiin	C_36_H_30_O_16_	717.14557	717.14622	−0.9	475.1037; 339.051; 519.0935; 476.1072; 365.0666	PAD
**45**	13.08	Dehydroxyl-rosmarinic acid-glucoside	C_24_H_26_O_12_	505.13461	505.13508	−0.9	161.0235; 323.0773; 181.0498; 179.0342; 324.0809	PAD
**46**	13.40	Apigenin-7-glucuronide	C_21_H_18_O_11_	445.07709	445.07747	−0.8	269.0456; 113.0231; 270.049; 85.0281; 59.0125	FV
**47**	13.46	Syringetin-glucoside	C_23_H_24_O_13_	507.11387	507.11422	−0.7	345.0616; 330.0382; 346.065; 331.0416; 315.0149	FV
**48**	13.82	Tuberonic acid hexoside	C_18_H_28_O_9_	387.16551	387.16587	−0.9	89.0230; 59.0125; 112.9843; 71.0125; 113.0232	OA
**49**	13.96	Rosmarinic acid	C_21_H_18_O_11_	359.07670	359.07689	−0.5	161.0235; 72.9917; 179.0341; 135.044; 197.045	PAD
**50**	14.08	Azelaic acid	C_9_H_16_O_4_	187.09704	187.09638	3.5	97.0646; 123.0803; 57.0332; 125.0961; 95.0489	OA
**51**	14.64	Apigenin caffeoyl glucoside	C_30_H_26_O_13_	593.12952	593.13009	−1.0	431.0985; 311.0564; 413.0882; 293.0458; 432.102	FV
**52**	15.16	Hydramacroside A	C_28_H_36_O_12_	563.21286	563.21326	−0.7	387.1663; 175.0392; 388.1696; 563.2132; 193.0499	SI
**53**	15.34	Isorhamnetin	C_16_H_12_O_7_	315.05048	315.05074	0.8	300.0277; 112.9843; 136.987; 301.0312; 68.9943	FV
**54**	15.39	Ferulic acid	C_10_H_10_O_4_	193.05009	193.04959	2.6	133.0284; 161.0235; 134.0354; 132.0207; 137.0236	PAD
**55**	15.96	4-Hydroxybenzoic acid	C_7_H_6_O_3_	137.02387	137.02282	7.6	93.0332; 65.0383; 94.0366; 75.0226; 66.0416	PAD
**56**	16.65	Unknown	C_17_H_30_O_8_	361.18625	361.18662	−1.0	68.9942; 112.9842; 161.0230; 346.1458; 101.0224	-
**57**	17.04	Luteolin	C_15_H_10_O_6_	285.03992	285.04008	−0.6	133.0283; 285.0405; 151.0026; 175.0392; 107.0126	FV
**58**	17.10	Luteone 7-glucoside	C_26_H28O11	515.15534	515.15563	−0.6	355.1188; 267.1034; 267.1394; 112.9843; 311.0934	FV
**59**	17.27	Unknown	C_20_H_18_O_6_	353.10252	353.10282	−0.8	247.1127; 265.087; 245.0968; 291.1024; 221.0970	-
**60**	17.49	Methyl rosmarinate	C_19_H_18_O_8_	373.09235	373.09270	−0.9	135.0440; 179.0342; 174.9552; 146.9602; 136.0474	PAD
**61**	17.64	Spinacetin	C_17_H_14_O_8_	345.06105	345.06136	−0.9	315.0149; 215.0344; 287.0198; 316.0181; 330.0383	FV
**62**	17.92	Trihydroxy-octadecadienoic acid	C_18_H_32_O_5_	327.21715	327.21740	−0.7	171.1018; 85.0281; 137.0961; 57.0332; 69.0332	FA
**63**	17.93	Unknown	C_20_H_16_O_6_	351.08687	351.08702	−0.4	281.0455; 219.081; 245.0966; 261.0921; 247.0758	-
**64**	18.06	Salvianolic acid F	C_17_H_14_O_6_	313.07122	313.07146	−0.8	161.0235; 133.0283; 162.0268; 123.0439; 151.039	PAD
**65**	18.18	Salvicanaric acid methyl ester	C_20_H_28_O_5_	347.18585	347.18607	−0.6	347.1865; 348.1899; 303.1604; 329.1759; 304.1631	TP
**66**	18.44	Salvianolic acid F	C_17_H_14_O_6_	313.07122	313.07143	−0.7	161.0234; 133.0283; 123.044; 151.0391; 162.0268	PAD
**67**	18.61	Unknown	C_21_H_22_O_8_	401.12365	401.12389	−0.6	266.9768; 401.1448; 121.7044; 191.5617; 214.7006	-
**68**	19.11	Trihydroxyoctadecenoic acid	C_18_H_34_O_5_	329.23280	329.23304	−0.7	211.1335; 171.1018; 229.1442; 112.9843; 183.1383	FA
**69**	19.21	Salvianolic acid F	C_17_H_14_O_6_	313.07122	313.07142	−0.6	161.0235; 133.0284; 162.0268; 123.044; 151.0391	PAD
**70**	19.38	Apigenin	C_15_H_10_O_5_	269.04500	269.04522	−0.8	117.0333; 151.0027; 269.0457; 149.0234; 107.0126	FV
**71**	19.60	Hydroxycarnosic acid	C_20_H_28_O_5_	347.18585	347.18598	−0.4	273.186; 317.1759; 274.1902; 271.1705; 245.1907	TP
**72**	19.73	Jaceosidin	C_17_H_14_O_7_	329.06613	329.06633	−0.6	299.0198; 313.0355; 300.0232; 314.0423; 271.0246	FV
**73**	19.93	Trihydroxy-dimethoxyflavone	C_17_H_14_O_7_	329.06613	329.06633	−0.6	299.0198; 313.0356; 300.0232; 314.0422; 285.0401	FV
**74**	20.40	Gibberellin A5 methyl ester	C_20_H_24_O_5_	343.15458	343.15473	0.5	343.1551; 271.0978; 344.1584; 218.058; 275.0927	TP
**75**	20.61	Carnosol isomer	C_20_H_26_O_4_	329.17528	329.17563	1.0	314.1526; 299.0198; 329.1758; 298.1214; 316.1317	TP
**76**	20.82	Hydroxyrosmadial	C_20_H_24_O_6_	359.14947	359.14967	−0.6	359.1503; 315.1602; 360.1537; 316.1635; 329.1399	TP
**77**	21.08	Trihydroxy-trimethoxyflavone	C_18_H_16_O_8_	359.07670	359.07703	−0.9	329.0305; 314.0071; 330.0338; 311.0201; 315.0106	FV
**78**	21.26	Rosmadial isomer	C_20_H_24_O_5_	343.15458	343.15473	0.5	343.1552; 328.1319; 344.1586; 313.1446; 298.1207	TP
**79**	21.38	Carnosic acid isomer	C_20_H_28_O_4_	331.19094	331.19132	−1.1	331.1916; 299.1653; 331.1586; 246.0897; 287.2019	TP
**80**	22.01	Hispidulin	C_16_H_12_O_6_	299.05557	299.05584	−0.9	284.0327; 285.0361; 256.0375; 299.056; 133.0283	FV
**81**	22.58	Cirsimaritin	C_17_H_14_O_6_	313.07122	313.07157	−1.1	283.0249; 284.0283; 297.0406; 255.0299; 163.0027	FV
**82**	22.66	Salvinal	C_20_H_20_O_6_	355.11817	355.11842	−0.7	355.1189; 356.1223; 235.0762; 325.0719; 201.0551	BF
**83**	22.93	Rosmaridiphenol isomer	C_20_H_28_O_3_	315.19602	315.19623	−0.6	315.1966; 283.1701; 112.9843; 68.9944; 230.0942	TP
**84**	22.97	Rosmadial derivative	C_20_H_26_O_5_	345.17020	345.17103	−2.4	345.1707; 314.0386; 346.1743; 171.1014; 315.0402	TP
**85**	23.29	Rosmadial isomer	C_20_H_24_O_5_	343.15458	343.15480	0.7	330.1474; 300.1368; 299.1653; 343.155; 315.1601	TP
**86**	23.36	Hydroxycarnosic acid	C_20_H_28_O_5_	347.18585	347.18584	0.01	332.1534; 303.1967; 302.1427; 304.2001; 347.1776	TP
**87**	23.38	Rosmadial derivative	C_20_H_26_O_5_	345.17020	345.17046	−0.7	330.1473; 300,1397; 331.1508; 315.0402; 301,1402	TP
**88**	23.60	Dihydroxy-trimethoxyflavone	C_18_H_16_O_7_	343.08178	343.08215	−1.1	313.0356; 298.012; 314.039; 193.0136; 299.0153	FV
**89**	23.75	FA 18:4+2O	C_18_H_28_O_4_	307.19094	307.19122	−0.9	119.0854; 97.0645; 137.096; 65.0383; 125.0959	FA
**90**	23.84	Hydroperoxyoctadecatrienoic acid	C_18_H_30_O_4_	309.20659	309.20698	−1.3	99.0802; 209.1177; 171.1018; 57.0332; 137.0963	FA
**91**	24.04	Dihydroxyoctadecadienoic acid	C_18_H_32_O_4_	311.22224	311.22258	−1.1	223.1700; 87.0437; 57.0333; 224.1735; 85.0281	FA
**92**	24.15	Carnosol isomer	C_20_H_26_O_4_	329.17528	329.17559	0.9	329.1759; 330.1793; 112.9843; 314.1518; 299.0200	TP
**93**	24.19	Acacetin/Genkwanin	C_16_H_12_O_5_	283.06065	283.06089	−0.8	268.0378; 269.0411; 240.0421; 117.0332; 239.0345	FV
**94**	24.23	Hydroxycarnosic acid	C_20_H_28_O_5_	347.18585	347.18620	−1.0	347.1864; 348.19; 331.1514; 303.1968; 243.1754	TP
**95**	24.26	Carnosol isomer	C_20_H_26_O_4_	329.17528	329.17563	1.03	329.1759; 330.1793; 112.9843; 314.1518; 299.02	TP
**96**	24.77	Rosmadial isomer	C_20_H_24_O_5_	343.15455	343.15486	−0.9	343.1551; 299.1656; 269.1182; 328.1314; 315.1611	TP
**97**	24.88	Carnosol isomer	C_20_H_26_O_4_	329.17528	329.17557	0.8	329.1759; 314.1525; 330.1793; 285.186; 315.1558	TP
**98**	24.90	Palmitoyl-sulfoquinovosyl glycerol	C_25_H_48_O_11_S	555.28391	555.28420	−0.5	555.2846; 556.2879; 225.007; 80.9637; 299.0440	FA
**99**	25.11	Hydroperoxyoctadecatrienoic acid	C_18_H_30_O_4_	309.20659	309.20692	−1.1	96.9588; 309.174; 125.0959; 171.1015; 79.9560	FA
**100**	25.30	GibberellinA24	C_20_H_26_O_5_	345.17020	345.17054	−1.0	257.1911; 81.0332; 301.1811; 259.1341; 283.1706	TP
**101**	25.35	Rosmanol	C_20_H_26_O_5_	345.1702	345.1704	0.6	283.1704; 330.1473; 315.1964; 284.1736	TP
**102**	25.42	Carnosol isomer	C_20_H_26_O_4_	329.17529	329.17563	−1.0	329.176; 69.0332; 330.1794; 285.1859; 287.2019	TP
**103**	25.75	Taxodione	C_20_H_26_O_3_	313.18038	313.18078	1.3	298.1573; 299.1608; 313.1809; 314.1844; 297.1488	TP
**104**	26.24	Hydroxy-deoxocarnosol	C_20_H_28_O_4_	331.19094	331.19116	−0.7	331.1916; 287.1654; 332.1952; 313.1812; 288.1686	FA
**105**	26.47	Rosmaridiphenol isomer	C_20_H_28_O_3_	315.19602	315.19618	−0.5	315.1967; 79.9559; 244.1103; 300.1732; 299.1653	TP
**106**	26.51	13-Hydroxy-9.11-octadecadienoic acid/13-HODE	C_18_H_32_O_3_	295.22728	295.22749	0.6	171.102; 277.2174; 195.1389; 295.2286; 113.096	FA
**107**	26.58	Hydroxy-deoxocarnosol	C_20_H_28_O_4_	331.19094	331.19134	−1.2	331.1915; 298.1574; 332.195; 285.1859; 270.1624	TP
**108**	26.62	Carnosol isomer	C_20_H_26_O_4_	329.17528	329.17560	0.9	301.1810; 302.1843; 286.1575; 329.1757; 271.1337	TP
**109**	26.64	Rosmaridiphenol isomer	C_20_H_28_O_3_	315.19598	315.19618	0.5	315.1967; 316.2000; 285.1861; 79.956; 286.1894	TP
**110**	26.70	Epirosmanol	C_20_H_26_O_5_	345.17020	345.17045	−0.7	286.1576; 245.1910; 273.1860; 289.1809; 287.1613	TP
**111**	26.75	Sugiol	C_20_H_28_O_2_	299.20108	299.20142	1.0	299.2018; 300.2051; 227.1073; 228.1119; 283.1698	TP
**112**	26.77	Rosmaridiphenol isomer	C_20_H_28_O_3_	315.19602	315.19622	−0.6	315.1967; 316.2001; 297.1861; 241.1231; 272.1420	TP
**113**	26.95	Carnosol isomer	C_20_H_26_O_4_	329.17528	329.17560	0.9	314.1527; 329.176; 315.1559; 330.1795; 299.0203	TP
**114**	27.06	Carnosol isomer	C_20_H_26_O_4_	329.17528	329.17558	0.9	314.1527; 315.1559; 329.1759; 299.0201; 330.1793	TP
**115**	27.28	Carnosol isomer	C_20_H_26_O_4_	329.17528	329.17560	0.9	329.1759; 330.1792; 314.1523; 313.1441; 299.1297	TP

* Classes: AA—amino acids; BF—benzofurans; FA—fatty acids; FV—flavonoids; OA—organic acids; P—purines/pyrimidines; PAD—phenolic acid derivatives; S—saccharides; SI—secoiridoids; TP—terpenoids.

**Table 2:** Average content of rosmarinic acid (mg/100 g DW ± SD) performed by HPLC-DAD analysis in seed, sprout, leaf, flower and herb extracts of *S. hispanica*.

Plant Material	Content (mg/100 g DW) ± SD
Seeds	127.25 ± 0.03
Sprouts	134.27 ± 0.04
Leaves	198.53 ± 0.18
Flowers	149.45± 0.03
Herb	185.12 ± 0.02

**Table 3:** The antibacterial and antifungal activities of seeds, sprout, leaf, root and herb extract *S. hispanica*.

Microorganisms	Whole Seeds *		Ground Seeds		Sprouts		Leaves		Herb		Roots *		Standard Drug (mg/L)	
	MIC	MBC/MFC	MIC	MBC/MFC	MIC	MBC/MFC	MIC	MBC/MFC	MIC	MBC/MFC	MIC	MBC/MFC	MIC	MBC/MFC
**Gram-positive bacteria**	mg/mL												**Vancomycin**	
*S. aureus* ATCC 25923	0.625	0.625	1.25	1.25	2.5	2.5	0.625	0.625	2.5	2.5	1.25	1.25	0.98	0.98
*S. aureus* ATCC BAA 1707	5	2.5	>10	10	5	5	1.25	1.25	5	5	2.5	>5	0.98	0.98
*S. epidermidis* ATCC 12228	5	>5	2.5	5	2.5	>10	0.313	0.313	2.5	2.5	2.5	>5	0.98	0.98
*M. luteus* ATCC 10240	0.07	0.625	0.15	1.25	0.625	1.25	1.25	1.25	2.5	5	0.625	5	0.12	0.12
*B. cereus* ATCC 10876	0.31	>5	0.625	>10	0.625	>10	1.25	>10	5	>10	1.25	>5	1.95	3.9
*E. faecalis* ATCC 29212	5	>5	1.25	>10	1.25	>10	1.25	5	5	>10	>5	>5	0.98	1.95
**Gram-negative bacteria**	mg/mL												**Ciprofloxacin**	
*S.* Typhimurium ATCC 14028	>5	>5	>10	>10	10	>10	5	>10	10	>10	>5	>5	0.061	0.06
*E. coli* ATCC 25922	>5	>5	>10	>10	10	>10	5	>10	>10	>10	>5	>5	0.015	0.08
*P. mirabilis* ATCC 12453	>5	>5	>10	>10	10	>10	2.5	2.5	5	>10	>5	>5	0.030	0.03
*K. pneumoniae* ATCC 13883	>5	>5	>10	>10	10	>10	10	>10	10	>10	>5	>5	0.122	0.24
*P. aeruginosa* ATCC 9027	>5	>5	10	>10	10	>10	5	>10	5	>10	>5	>5	0.488	0.98
**Fungi**	mg/mL												**Nystatin**	
*C. glabrata* ATCC 90030	5	5	10	>10	10	>10	10	>10	10	>10	0.625	5	0.48	0.48
*C. albicans* ATCC 102231	0.07	5	10	>10	2.5	>10	5	>10	10	>10	1.25	5	0.24	0.48
*C. parapsilosis* ATCC 22019	0.003	5	10	>10	0.625	10	10	10	10	10	0.31	5	0.24	0.48

MIC—minimal inhibitory concentration, MBC—minimal bacteridical concentration, MFC—minimal fungicidal concentration [mg/mL]. * in concentration range from 5 to 0.003 mg/mL.

## Data Availability

Not applicable.

## Sample Availability

Samples are not available from the authors.
